# STC1 promotes cell apoptosis via NF-κB phospho-P65 Ser536 in cervical cancer cells

**DOI:** 10.18632/oncotarget.17641

**Published:** 2017-05-05

**Authors:** Xi Pan, Binyuan Jiang, Jianhao Liu, Juan Ding, Yuehui Li, Ruili Sun, Li Peng, Changfei Qin, Shujuan Fang, Guancheng Li

**Affiliations:** ^1^ The Key Laboratory of Carcinogenesis of The Chinese Ministry of Health and The Key Laboratory of Carcinogenesis and Cancer Invasion of The Chinese Ministry of Education, Xiangya Hospital, Central South University, Changsha 410008, China; ^2^ Cancer Research Institute, Central South University, Changsha 410078, China; ^3^ Xiangya Third Hospital, Central South University, Changsha 410078, China; ^4^ School of Pharmaceutical Sciences of Central South University, Changsha 410078, China

**Keywords:** stanniocalin-1 (STC1), cell apoptosis, cervical cancer, NF-κB, phospho-P65 (Ser536)

## Abstract

Stanniocalin-1 (STC1) is a secreted glycoprotein hormone and involved in various types of human malignancies. Our previous studies revealed that STC1 inhibited cell proliferation and invasion of cervical cancer cells through NF-κB P65 activation, but the mechanism is poorly understood. In our studies, we found overexpression of STC1 promoted cell apoptosis while silencing of STC1 promoted cell growth of cervical cancer. Phospho-protein profiling and Western blotting results showed the expression of NF-κB related phosphorylation sites including NF-κB P65 (Ser536), IκBα, IKKβ, PI3K, and AKT was altered in STC1-overexpressed cervical cancer cells. Moreover, PI3K inhibitor LY294002, AKT-shRNA and IκBα-shRNA could decrease the protein content of phospho-P65 (Ser536), phospho-IκBα, phospho-AKT and phospho-IKKβ while increasing the level of P65 compared to STC1 overexpression groups in cervical cancer cells. Also, PI3K inhibitor LY294002, AKT-shRNA and IκBα-shRNA elevated the percentage of apoptosis and suppressed the G1/S transition in those cells. Additionally, STC1 level was decreased in cervical cancer, especial in stage II and III. The results of immunohistochemistry for the cervical cancer microarray showed that a lower level of STC1, phospho-PI3K and P65 protein expression in tumor tissues than that in normal tissues, and a higher level of phospho-P65 protein expression in tumor tissues, which is consistent with the results of the Western blotting. These data demonstrated that STC1 can promote cell apoptosis via NF-κB phospho-P65 (Ser536) by PI3K/AKT, IκBα and IKK signaling in cervical cancer cells. Our results offer the first mechanism that explains the link between STC1 and cell apoptosis in cervical cancer.

## INTRODUCTION

As a common gynecological malignancy, cervical cancer is the third most fatal cancer in women worldwide [1, 2], especially in developing countries [3]. Despite of the widely-used treatment of cervical cancer involving radical surgery, radiotherapy and chemotherapy, there still around 40% of patients overall will develop persistent/recurrent/metastatic disease. To this day the pathogenesis of cervical cancer is largely unknown, so the underlying mechanisms for cervical cancer and progression are still under investigation.

Stanniocalcin-1 (STC1) is a secreted glycoprotein hormone [4], which was first identified as a hypocalcaemia hormone functioning importantly for the maintenance of calcium homeostasis in teleost fish [5, 6]. Recent studies found that STC1 is expressed abundantly in a variety of mammalian tissues including kidney [7], heart [8], lung [9], ovary [10], brain [11], muscular and skeletal tissues [12]. STC1 is highly conserved during evolution, and is implicated in several physiologies and pathologies, such as pregnancy [13], angiogenesis [14], inflammation and apoptosis [15]. Although most of studies have focused on the calcium-regulating functions of STC1, increasing evidence suggests that STC1 may also play a major role in carcinoma. High expression of STC1 was frequently detected in human tumor samples of hepatocellular carcinoma (HCC) [16], colorectal cancer [17], lung adenocarcinoma [9], breast cancer [18, 19] and thyroid carcinomas [20], however, low expression of STC1 was found in tumor-derived ovarian epithelial cells [21]. Our previous studies have shown that STC1 is on the decrease in cervical cancer cells for the first time, and that it suppresses cellular multiplication and metastasis of cervical cancer cells likely through NF-κB P65 protein [22]. However, the role and molecular mechanism of STC1 in the cell apoptosis of cervical cancer remain to be fully elucidated.

Our previous studies have shown that NF-κB P65 protein may directly bind to the promoter of STC1 and activate the expression of STC1 in cervical cancer cells [22]. The transcription factor NF-κB was found in 1986 to be a nuclear factor that binds to the enhancer element of the immunoglobulin kappa light-chain of activated B cells (NF-κB). NF-κB plays a critical role in diverse human physiological processes and pathologies [23]. It has been identified that five members exist for the transcription factor NF-κB: RelA (P65), RelB and c-Rel, and the precursor proteins NF-κB1 (p105) and NF-κB2 (p100), which are processed into p50 and p52, respectively [24, 25]. RelA/P65 is mainly phosphorylated at the amino-terminal REL homology domain (RHD, including Ser376 and Ser311) and at the the transcriptional activation domain (TAD) of the carboxy-terminus (such as Ser539 and Ser536). Yet, the specific phosphorylation site of NF-κB P65 that is involved in the anti-apoptotic effect of STC1 in cervical cancer cells is unclear.

In this study, we reported a molecular mechanism of STC1 regulating cell apoptosis of cervical cancer, which was through regulating cell apoptosis via NF-κB phospho-P65 (Ser536) by PI3K/AKT, IκBα and IKK signaling. Our findings provide a novel insight for STC1 as a target or biomarker in the therapy and prevention of cervical cancer.

## RESULTS

### Expression of STC1 in cervical cancer is associated with tumor stage

**Figure 1:**
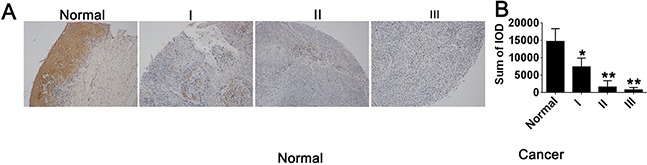
STC1 expression was associated with tumor stage in cervical cancer The antigens/proteins of STC1, P65, phospho-P65 (P-P65), PI3K, phospho-PI3K (P-PI3K) were in cervical normal and cancer tissues. **(A)** The expression of STC1 in different stages of cervical cancer tissues and Normal tissues, especial low expression in stage II and III (*p*=0.034). Normal, I, II, III represent the different cervical cancer stages. **(B)** The expression of STC1 in different stages of cervical cancer tissues and Normal tissues. IOD means integral optical density, representing the expression level of STC1 in each tissue sample, is calculated with image-pro plus.

To explore the precise role of STC1 in cervical cancer diagnosis and prognosis, we examined the expression of STC1 in cervical cancer tissues and normal tissues by immunohistochemistry. The results showed that STC1 was mainly localized in the nucleus of cervical cancer cells and was lower expression in cervical tumor tissues than normal tissues (Figure 1A). The results of IOD analysis revealed that the level of STC1 in cervical cancer was significantly associated with tumor stage (*p***=**0.034, Figure 1B), but did not differ depending on patient age (<45 years or ≥45 years, *p***=**0.237; Supplementary Table 1), implying that STC1 might act as a potential cancer biomarker.

### STC1 inhibited cell growth of cervical cancer cells

**Figure 2:**
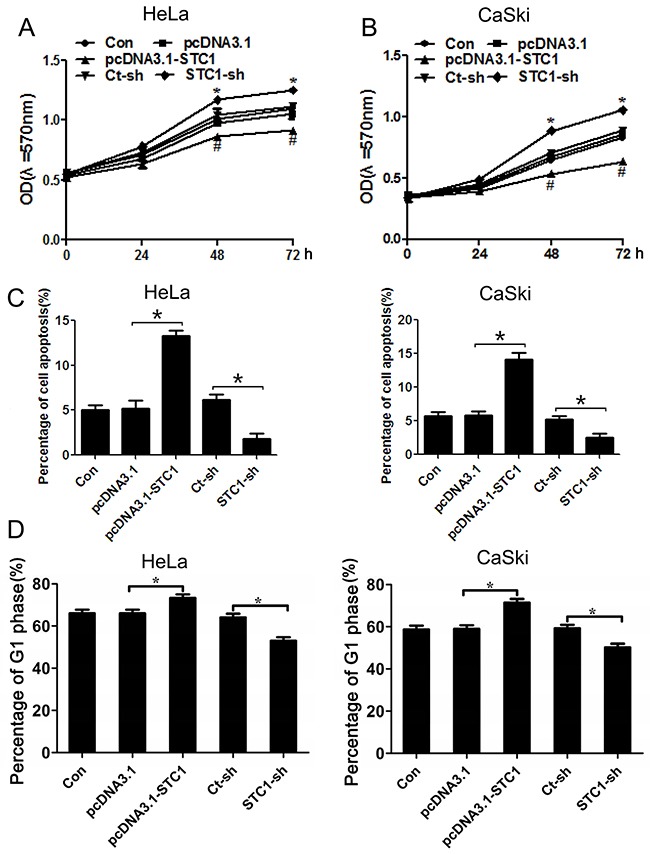
STC1 inhibited cell growth of cervical cancer cells MTT assay shows STC1 suppressed cell proliferation in cervical cancer HeLa and CaSki cells **(A, B)**, and Annexin V-FITC /PI staining shows STC1 promoted cell apoptosis in cervical cancer HeLa and CaSki cells **(C)**. n=3, *p<0.05. Flow cytometry analysis reveals STC1 arrested G1 phase of cell cycle in cervical cancer HeLa and CaSki cells **(D)**. n=3, *p<0.05.

To further verify the function of STC1 in cervical cancer cells, cell growth was detected by MTT assays and annexin V-FITC/PI staining. Firstly, we constructed the overexpression and silencing of STC1 vectors (pcDNA3.1-STC1 and STC1-sh), then tested the efficiency of STC1 vectors by RT-PCR and Western blotting assays. Results displayed that the mRNA and protein levels of STC1 were increased when HeLa and CaSki cells were treated with pcDNA3.1-STC1 compared with pcDNA3.1 empty vector, but it was reduced when the cells were treated with STC1-sh vector compared with Ct-sh vector (Supplementary Figure 1), suggesting the vectors containing pcDNA3.1-STC1 and STC1-sh were constructed successfully. MTT assay showed that overexpression of STC1 dramatically suppressed cell growth while silencing of STC1 promoted cell growth of HeLa and CaSki cells (Figure 2). Moreover, the results of flow cytometry showed that overexpression of STC1 can accelerate cell apoptosis and silencing of STC1 can restrain cell apoptosis of HeLa and CaSki cells (Figure 2C and Supplementary Figure 2). In addition, overexpression of STC1 can arrest G1/S transition, but silencing of STC1 can accelerate G1/S transition of HeLa and CaSki cells (Figure 2D and Supplementary Figure 2). These results suggested that STC1 can inhibit cell growth of cervical cancer HeLa and CaSki cell lines.

### Alteration of phosphorylation sites in NF-κB signaling

To investigate whether NF-κB was involved in the pro-apoptotic effect of STC1 or not in cervical cancer, we analyzed Phospho-protein profiling in CaSki/STC1 cells and CaSki/NC cells via phospho-protein antibody array designed for NF-κB signaling pathway. Several proteins were vital for cellular apoptosis with their significant alteration. These apoptosis-related proteins included AKT, IκBα, IKK, JNK, P65, PI3K, CK2, COT, PKR and TAK1 (Supplementary Table 2). We further verified these proteins for their expression and phosphorylation changes induced by STC1 overexpression using Western blotting.

**Figure 3:**
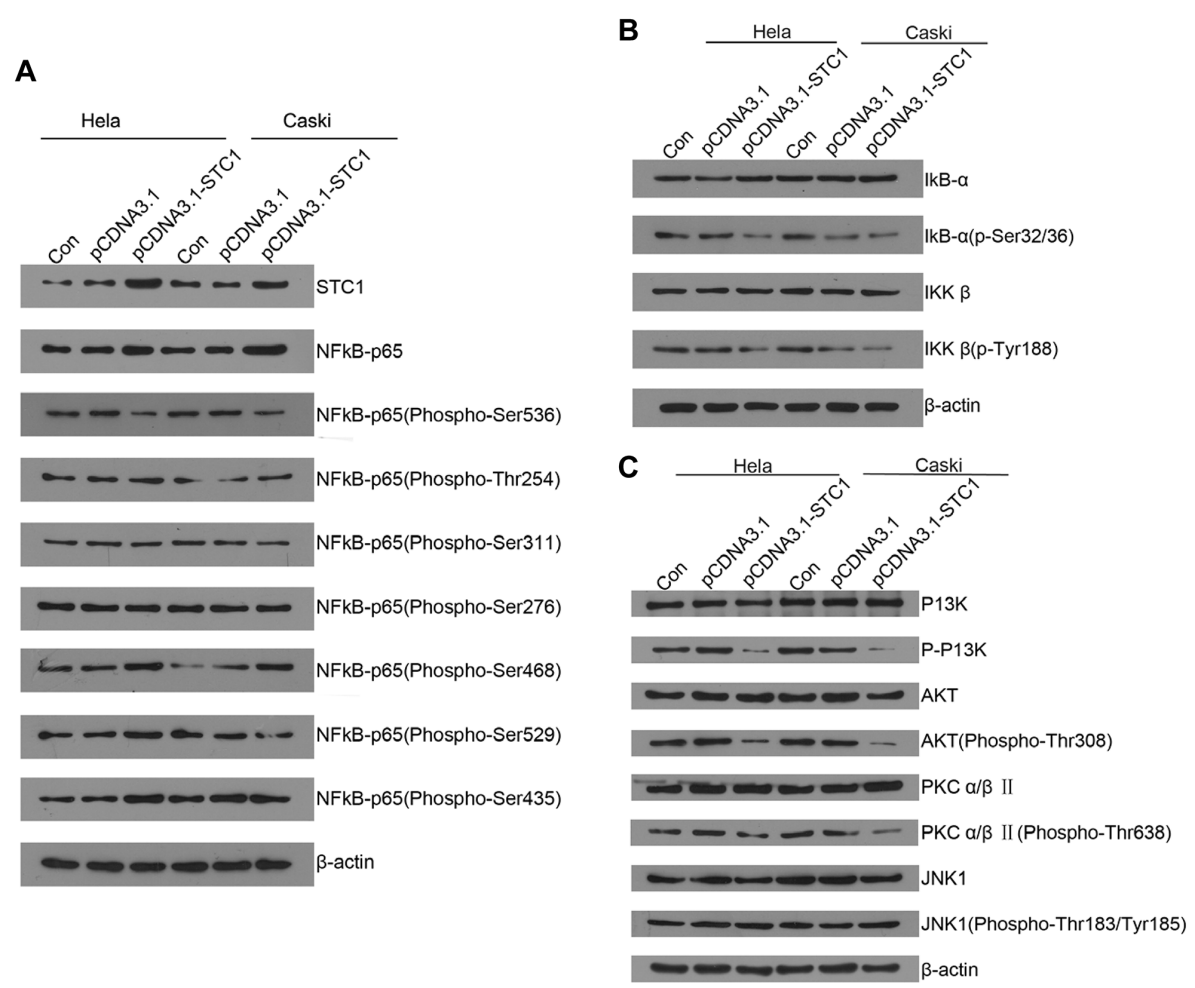
The protein and phosphorylation levels of essential proteins in STC1 overexpressed cervical cancer cells **(A)** The protein levels of NF-κB P65 and its exact phosphorylation site in STC1 overexpressed cervical cancer HeLa and CaSki cells were detected by Western blotting. **(B)** The protein and phosphorylation levels of IκBα and IKKβ in STC1 overexpressed cervical cancer HeLa and CaSki cells were compared using Western blotting. **(C)** The protein levels of PI3K, AKT, PKCα/β and JNK1 in STC1 overexpressed cervical cancer HeLa and CaSki cells were tested by Western blotting. n=3, *p<0.05.

The results showed that overexpression of STC1 significantly down-regulated the phosphorylation of IκBα (Ser32/36) and IKKβ protein, phosphorylation of P65 protein in Ser536, phosphorylation of PI3K and AKT protein, which indicated the NF-κB signaling pathway was activated by these signal pathways (Figure 3 and Supplementary Figure 3). In conclusion, STC1 may activate P65 to inhibit cell growth through PI3K/AKT signaling pathway in Hela and Caski cells.

### STC1 downregulated phospho-P65 (Ser536) by PI3K/AKT, IκBα and IKK signaling

**Figure 4:**
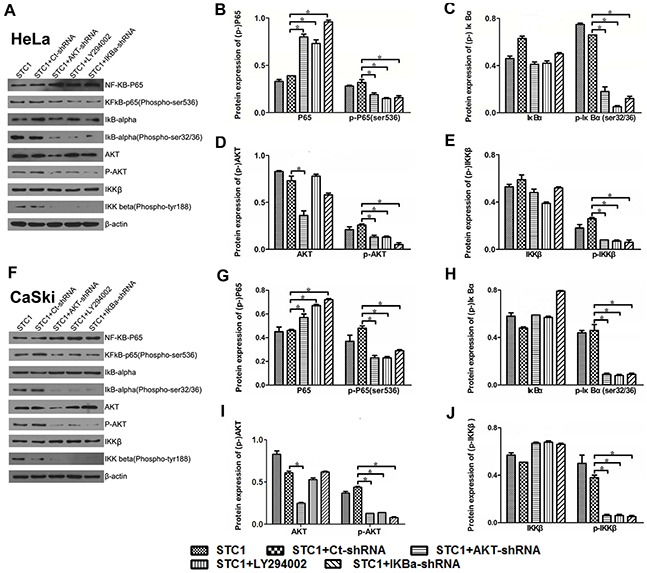
The protein and phosphorylation levels of key proteins in cervical cancer cells Western blotting shows the protein and phosphorylation levels of NF-κB phospho-P65 (Ser536), IκBα, AKT and IKKβ with the treatment of AKT-shRNA, PI3K inhibitor LY294002 and IκBα-shRNA in STC1 overexpressed HeLa **(A-E)** and CaSki cells **(F-J)**. n=3, *p<0.05.

To determine the role of PI3K/AKT inhibitors LY294002 in Hela and Caksi cells, Supplementary Figure 4 showed LY294002 significantly inhibited the expression of AKT in a dose-dependent manner, and 10 μmol/L was selected for the related experiment. To further illuminate the relationship among STC1, PI3K/AKT, P65 (Ser536) and cell growth of Hela and Caski, we treated cells with PI3K inhibitor LY294002, AKT-shRNA and IκBα-shRNA in STC1 overexpressed cells, and detected the expression and phosphorylation levels of the signaling-related proteins in cervical cancer HeLa and CaSki cell lines. Firstly, we constructed some vectors containing AKT-shRNA and IκBα-shRNA vector, and tested their efficiency. It was displayed that the mRNA and protein levels of AKT and IκBα were reduced when HeLa and CaSki cells were respectively treated with AKT-shRNA and IκBα-shRNA vector (Supplementary Figure 5), suggesting the vectors containing AKT-shRNA and IκBα-shRNA were transfected effectively. Western blotting results showed that LY294002, AKT-shRNA and IκBα-shRNA significantly increased the protein expression of P65 and declined the protein expression of phospho-P65 (Ser536) in STC1 overexpressed HeLa (Figure 4A, 4B) and CaSki cells (Figure 4F, 4G). Additionally, LY294002, AKT-shRNA and IκBα-shRNA could decrease the protein content of phospho-IκBα and phospho-IKKβ in STC1 overexpressed HeLa (Figure 4A, 4C, 4E) and CaSki cells (Figure 4F, 4H, 4J) compared with the control cells. At the same time, LY294002, AKT-shRNA and IκBα-shRNA could decrease the protein content of phospho-AKT in STC1 overexpressed HeLa (Figure 4A, 4D) and CaSki cells (Figure 4F, 4I). These results demonstrated that the inhibition of PI3K/AKT signaling pathway can downregulate the expression of phospho-P65, phospho-IκBα and phospho-IKK expression in STC1 overexpression condition.

**Figure 5:**
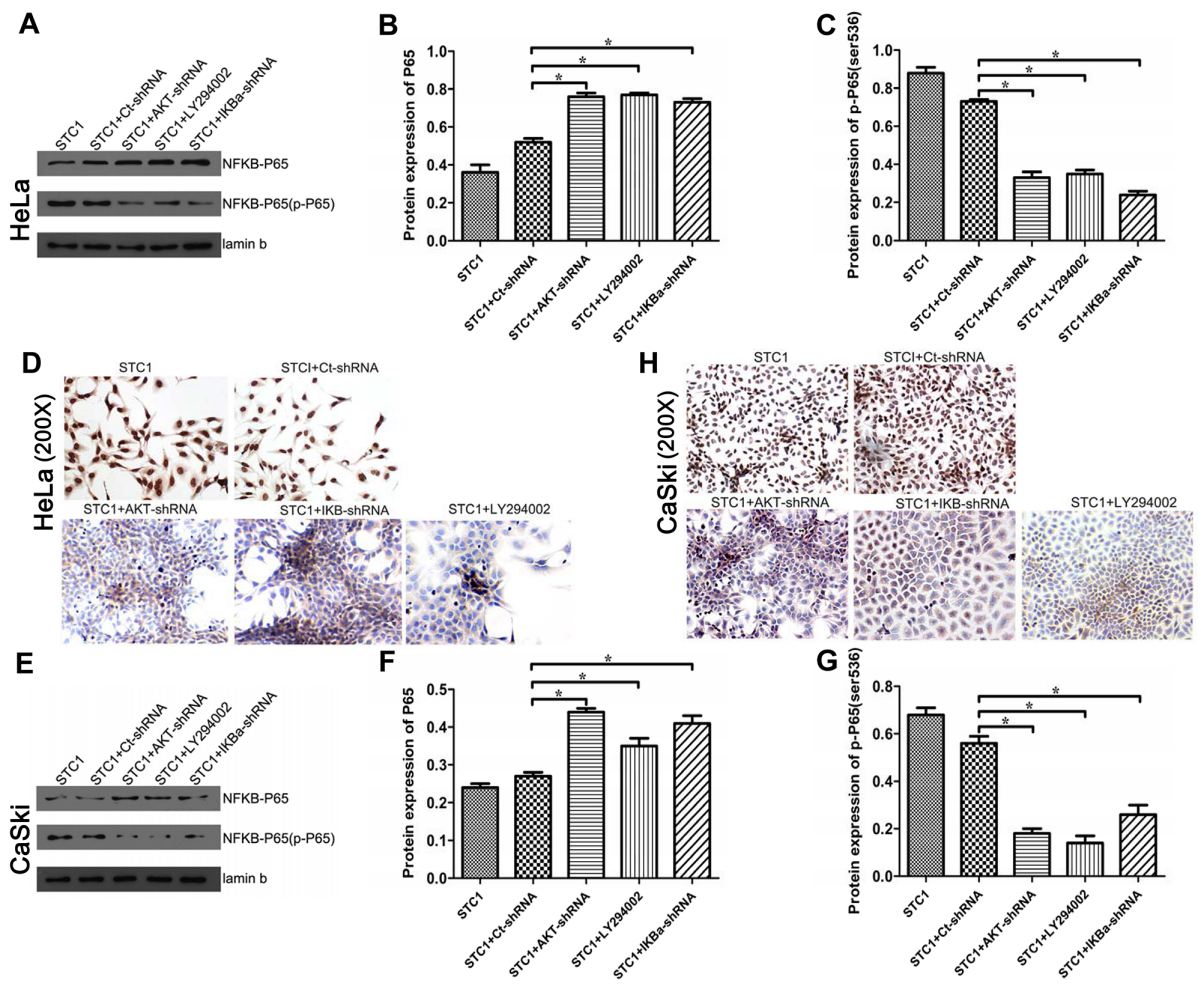
The nucleprotein and phosphorylation levels of P65/phospho-P65 (Ser536) in cervical cancer cells NF-κB P65 nucleprotein in STC1 overexpressed HeLa **(A, B)** and CaSki **(E, F)** cell lines treated with the AKT-shRNA, PI3K inhibitor LY294002 and IκBα-shRNA were detected by Western blotting. Phospho-P65 (Ser536) nucleprotein in STC1 overexpressed HeLa **(C, D)** and CaSki **(G, H)** cell lines treated with the AKT-shRNA, PI3K inhibitor LY294002 and IκBα-shRNA were detected by Western blotting and ICC. n=3, *p<0.05.

To further illustrate the involvement of PI3K/AKT, IκBα and IKK signaling in the regulation of phospho-P65 (Ser536), the nucleoprotein expression of P65 and phospho-P65 (Ser536) was detected using Western blotting and immunocytochemistry (ICC) after STC1 overexpressed HeLa and CaSki cells being treated with PI3K inhibitor LY294002, AKT-shRNA and IκBα-shRNA. Results revealed that PI3K inhibitor LY294002, AKT-shRNA and IκBα-shRNA dramatically increased the nucleoprotein content of NF-κB P65 while decreasing the protein level of phospho-P65 (Ser536) in STC1 overexpressed HeLa (Figure 5A-5C) and CaSki cells (Figure 5E-5G) compared to STC1 overexpressed groups via Western blotting. In addition, overexpression of STC1 significantly increased the protein expression of phospho-P65 in the cell nucleus, however PI3K inhibitor LY294002, AKT-shRNA and IκBα-shRNA decreased the level of phospho-P65 (Ser536) and the expression of phospho-P65 in cytoplasm in STC1 overexpressed HeLa (Figure 5D) and CaSki cells (Figure 5H) via ICC. These results aslo demonstrated that STC1 down-regulated phospho-P65 (Ser536) by PI3K/AKT, IκBα and IKK signaling.

### STC1 inhibited apoptosis by PI3K/AKT, IκBα and IKK signaling

**Figure 6:**
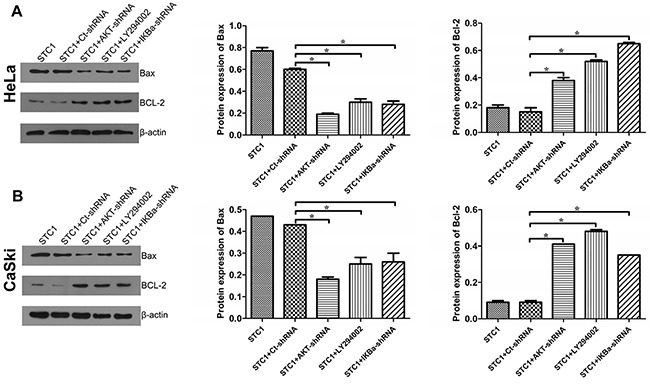
The protein levels of Bax/Bcl-2 in cervical cancer cells The protein levels of pro-apoptotic Bax and anti-apoptotic Bcl-2 in STC1 overexpressed HeLa **(A)** and CaSki **(B)** cells treated with the AKT-shRNA, PI3K inhibitor LY294002 and IκBα-shRNA were tested by Western blotting. n=3, *p<0.05.

Cancer is initiated when there is an upregulation of anti-apoptotic genes (including Bcl-2) and downregulation of pro-apoptotic genes (containing Bax) [26]. Bax/Bcl-2 is essential for the cell apoptosis process in many cells including cervical cancer cells [26]. So we detected the regulatory effect of PI3K/AKT, IκBα and IKK signaling on the expression of apoptosis related genes Bax and Bcl-2, in order to make sure whether STC1 works through the PI3K/AKT signaling to regulate cell apoptosis of cervical cancer cells. It was showed that PI3K inhibitor LY294002, AKT-shRNA and IκBα-shRNA could decrease the protein content of Bax while increasing the level of Bcl-2 in STC1 overexpressed HeLa (Figure 6A) and CaSki (Figure 6B) cells by Western blotting.

**Figure 7:**
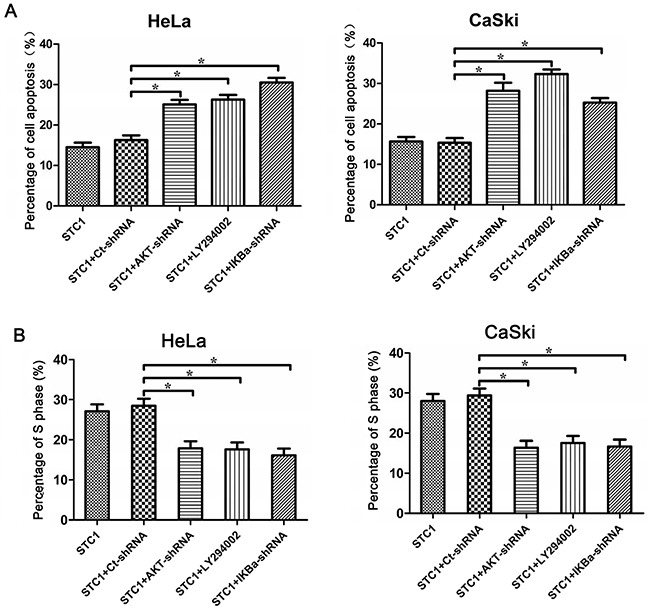
AKT, PI3K and IκBα inhibited apoptosis and promoted G1/S transition in STC1 overexpressed cervical cancer cells **(A)** Annexin V-FITC/PI staining shows the inhibition of AKT (AKT-shRNA), PI3K (PI3K inhibitor LY294002) and IκBα (IκBα-shRNA) promoted the cell apoptosis of cervical cancer HeLa and CaSki cells. **(B)** Annexin V-FITC /PI staining reveals the AKT-shRNA, PI3K inhibitor LY294002 and IκBα-shRNA suppress the S phase of cervical cancer HeLa and CaSki cells. n=3, *p<0.05.

In order to further demonstrate the involvement of PI3K/AKT signaling in cell apoptosis of cervical cancer cells, cell apoptosis and cell cycle were determined via Annexin V/PI staining and flow cytometry analysis, respectively. Results revealed that PI3K inhibitor LY294002, AKT-shRNA and IκBα-shRNA elevated the percentage of apoptosis in STC1 overexpressed HeLa and CaSki cells compared with the STC1 overexpressed group by Annexin V/PI staining (Figure 7A and Supplementary Figure 6). In addition, PI3K inhibitor LY294002, AKT-shRNA and IκBα-shRNA suppressed the G1/S transition in STC1 overexpressed HeLa and CaSki (Figure 7B and Supplementary Figure 6) cells. The above results showed that PI3K/AKT, IκBα and IKK signaling could involve the inhibitory effect of STC1 on cell proliferation in cervical cancer cells.

### Immunohistochemistry results were consistent with the western blotting

**Figure 8:**
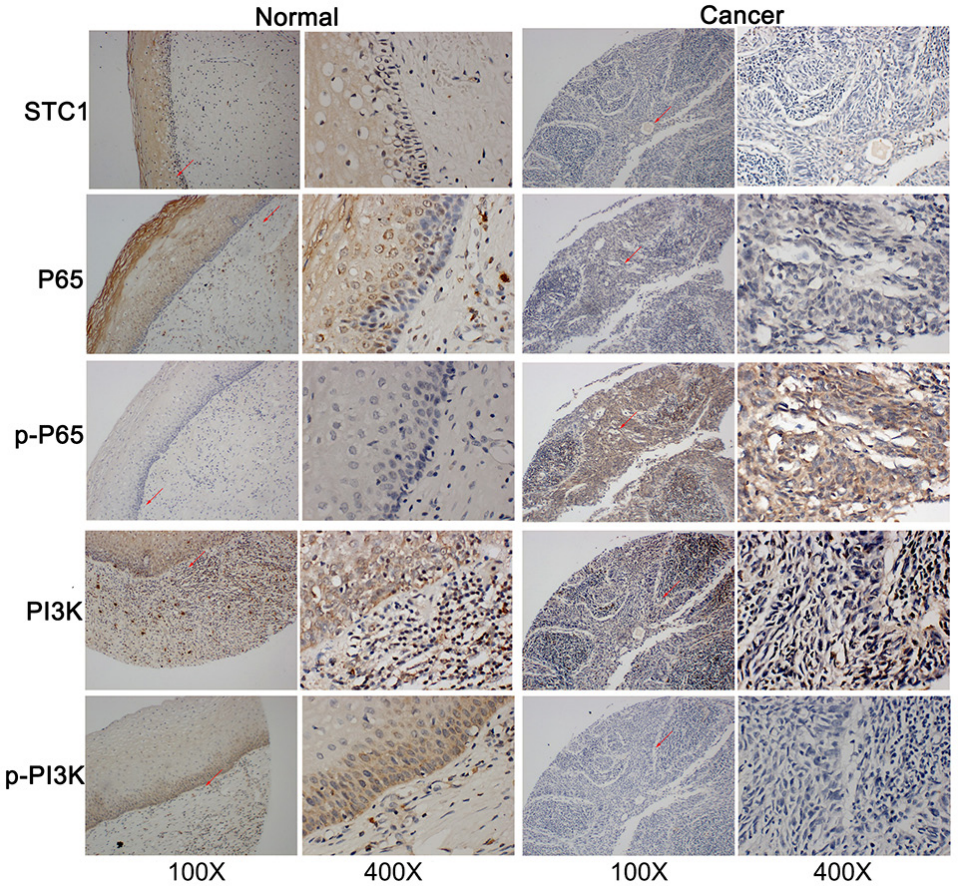
The antigens/proteins of STC1, P65, phospho-P65 (P-P65), PI3K, phospho-PI3K (P-PI3K) in cervical normal and cancer tissues The expression of STC1, P65, phospho-P65 (P-P65), PI3K, phospho-PI3K (P-PI3K) in cervical normal and cancer tissues detected by immunohistochemistry. All figures: immunohistochemistry, hematoxylin counterstain.

The results of immunohistochemistry for the cervical cancer microarray showed that the antigens of STC1 and phospho-PI3K were weakly detected in tumor tissues (*p*=0.008 and *p*=0.049, respectively), and the P65 antigen was too weak to be detected. However the phospho-P65 (Ser536) antigen was strong in tumor tissues (*p*=0.044). These data are consistent with the results of the Western blotting (Figure 8).

## DISCUSSION

In recent years, studies have indicated that STC1 plays a key role in cell proliferation and apoptosis. Some studies have suggested the anti-apoptosis effect of STC1, such as colorectal cancer [27, 28], breast cancer [19, 29], glioma tumor [11, 30], thyroid cancer [20, 31], ovarian cancer [32, 33] and so on. Others indicated the pro-apoptosis effects. A statistical and genetic analysis on the growth of hepatocellular carcinoma cells is a case in point [16]. We showed that STC1 could promote cell apoptosis and arrest G1/S transition, and STC1 promoted cell apoptosis via NF-κB phospho-P65 (Ser536) by PI3K/AKT, IκBα and IKK signaling in cervical cancer cells, thus having the potential to provide a novel direction for the therapy and prevention of cervical cancer. As a result, the role of STC1 may depend on the tissue specificity and condition specificity.

NF-κB activation is tightly modulated mainly through its localization. In resting cells, NF-κB proteins are kept in the cytoplasm in association with inhibitory IκB proteins including IκBα, IκBβ, and IκBε among which IκBα is the most abundant [34]. Our results indicated that IκBα could inhibit the expression of NF-κB P65 while enhancing the phosphorylation level of P65 (Ser536). In addition, stimulus-induced degradation of IκB proteins is initiated through phosphorylation by the IκB kinase (IKK) complex, which consists of two catalytically active kinases, IKKα and IKKβ, and the regulatory subunit IKKγ (NEMO) [24]. So we tested the regulation relationship between IKK and IκBα, showing that inhibiting the expression of IκBα decreases the phosphorylation level of IKKβ.

As mentioned above, NF-κB P65 is one of five members in the transcription factor NF-κB, which mainly phosphorylates at Ser311, Ser376 and Ser536 etc. Our previous studies showed that NF-κB P65 protein was likely to activate the expression of STC1 in cervical cancer cells [22]. As an important phosphorylation site of NF-κB P65, Ser536 is involved in regulation of transcriptional activity, nuclear localization and protein stability [35]. Ser536 in NF-κB P65 has been shown to trigger vast apoptosis in colon, breast and prostate and inhibit their tumor growth [36]. Our results suggest that NF-κB P65 phosphorylation at Ser536 is involved in anti-apoptotic effect of STC1 in cervical cancer cells. In addition, P65 Ser536 is the target of many kinases, including IκB, IKKs (α, β and ε) and ribosomal subunit kinase-1 (RSK1) [37, 38]. But none of them has been systematically studied in cervical cancer. Whether these kinases involved in phosphorylation of Ser536 with STC1 have a role in cervical cancer cells needs to be further analyzed. In this study, we found that STC1 overexpression lowered the phosphorylation level of NF-κB P65 (Ser536). The trend is in accordance with the previously reported studies showing that the decrease of phosphorylation level in Ser536 can promote cell apoptosis [36, 39]. In addition, there were no obvious changes in other sites. Therefore, STC1 is most likely to function as a regulator through the phosphorylation site Ser536.

Furthermore, we found that STC1 overexpression may inhibit malignant biological behavior via NF-κB P65 phospho-Ser536 through PI3K/AKT signaling. It was reported that IKKβ-mediated NF-κB P65 phospho-Ser536 required PI3K-AKT activity, which is elicited by cytokines as well as growth factors and thus represents an emerging node for crosstalk between the NF-κB P65 and PI3K-AKT pathways [24]. PI3K-AKT signaling pathway regulated the fundamental cellular functions and associated with cancer development and progression [40]. For example, it was shown to function through being regulated by inflammatory cytokines (IL-6, CCR9 and TLR3) during apoptosis, and involve in the pathogenesis of prostate cancer [41]. To further explore whether PI3K-AKT signaling was related to NF-κB P65, IκBα and IKKβ signaling in cervical cancer, we tested the expression changes of these signaling molecules after cervical cancer cells were treated with PI3K inhibitor LY294002 and AKT-shRNA vector. Our results demonstrated that inhibition of PI3K/AKT signaling pathway decreased phosphorylation level of signaling factor IκBα and IKKβ, and accelerated G1/S arrest and cell apoptosis.

In conclusion, our studies confirmed that the expression of STC1 in cervical cancer is associated with tumor stage. To promote cell apoptosis, STC1 down-regulates the content of NF-κB phospho-P65 (Ser536) in cervical cancer cells by PI3K/AKT, IκBα and IKK signaling in cervical cancer cells.

## MATERIALS AND METHODS

### Tissues microarray using immunohistochemistry

Tissue microarray (Auragene, Changsha, China) containing 80 cervical cancer specimens was prepared for immunochemistry. Antigen retrieval was performed in pH 6.0 citrate buffers, by using a pressure cooker at 104°C for 32 min with a 10 min bench cool down, followed by quenching with 3% H_2_O_2_ w/sodium azide for 15 min. After blocking in a serum-free protein block for 1 h, P65 antibody (1:100, Immunoway, Newark, USA), phospho-P65 antibody (1:100), PI3K antibody (1:100), phospho-PI3K antibody (1:100), STC1 antibody (1:100, santa cruz, Texas, USA) was incubated with the samples for overnight at 4°C, then the tissue microarray was detected with Dako Envision + HRP Labeled Polymer (Auragene, Changsha, China) for 30 min after incubating with chromogen DAB+ for 30 s and hematoxylin for 15 min. The sum of IOD of STC1 expression was determined by IPP6.0.

### Cell culture

Cervical cancer HeLa and CaSki cell lines were acquired from the American Type Culture Collection (ATCC, Maryland Rockefeller, USA). All these cells were cultured in RPMI-1640 medium containing 10% FBS and 1% antibiotic-antimycotic solution (100 U/mL penicillin and 100 μg/mL streptomycin). Cells were cultured in a 37°C incubator in a humidified atmosphere containing 5% CO_2_.

### Construction of STC1 overexpression vector

Primer for STC1 full length amplication were synthesized with Hind III and EcoR I restriction enzymes sites. The forward primer was 5′-CCCAAGCTT ATGCTCCAAAACTCAGCAGTGCT-3′ (Hind III), and the reverse primer was 5′-CCGGAATTCTTATGCACTCTCATGGGATGTGC-3′ (EcoR I). HeLa and CaSki cells genomic DNA were used as the template. The purified PCR product was double–enzyme digested and ligated into the eukaryotic expression vector pcDNA3.1 (+). HeLa and CaSki cells were subject to transfection with specific plasmids by Lipofectamine 2000 agent (Invitrogen, Carlsbad, USA); pcDNA3.1 (+) vector was acted as control.

### Construction of STC1-shRNA vector, AKT-shRNA vector and IκBα-shRNA vectors

The shRNA target sequence for human STC1 was 5′-TTAGTCCAGGAAGCAATAGTA-3′, for AKT-shRNA was 5′-GGAGTGTTAAGCGTTCAGTGA-3′, and for IκBα-shRNA was 5′-GGACTACCTGCAC TCGGAGAA-3′. The forward and reverse primer sequences of candidate targets are shown as Supplementary Table 3. The shRNA-annealed oligonucleotides were ligated into the vector pRNAT-U6.1/Neo encoding a small hairpin RNA directed against the target gene in Hela and CaSki cells to establish the these shRNA vectors (STC1-shRNA, AKT-shRNA and IκBα-shRNA) by T4 DNA ligase (Takara, Dalian, China). The constructs were verified by sequencue analysis (BGI, Tech, Shenzhen, China).

### MTT assay

Approximately 5×10^3^ cells/wellwere plated into 96-well plates (Costar, Corning, NY, USA). After culturing for 24/48/72 h, cells were treated with fresh serum-free medium and 10 μL/well MTT (Biosharp, Hefei, China) solution (10 mg/mL in PBS) according to the manufacturer's protocol. The 100 μL of DMSO was added to each well after the incubation for 4 h. After incubation at 37°C for 10 min, the absorbance was measured by microplate reader (Thermo, Waltham, MA, USA) in absorbance at 570 nm at room temperature.

### Annexin V/propidium iodide staining detected cell apoptosis

Apoptosis of HeLa and CaSki cell lines were determined via Annexin V-FLUOS staining Kit (Roche, Basel, Switzerland) following the treatment with pcDNA3.1, pcDNA3.1-STC1, Ct-sh and STC1-sh, or STC1. The cells were collected, washed twice with PBS, and were resuspended in 500 μL binding buffer. Then, cells were mixed with 5 μL Annexin-V-FITC and 5 μL PI in order. After being mixed, the cells were placed in the dark to react for 15 min at 15-25°C. Samples were analyzed within 1 h.

### Flow cytometry analysis determined cell cycle

HeLa and CaSki cells were harvested from 6-well plates after transfection. Harvested cells were washed, fixed, permeabilized, and suspended in 0.3 mL PBS containing PI (50 μg/mL) at 4°C for 30 min. The percentage of cells in the different stages was measured by Flow Cytometry (No. Moflo XDP, Beckman Coulter, California, USA) and was evaluated using FlowJO software.

### STC1-associated phospho-protein profiling

Cell lysates were acquired from STC1 overexpression group (CaSki/STC1) and the corresponding control group (CaSki/NC) in cervical cancer cell lines, and were analyzed by using a human NF-κB Phospho Antibody Array (PNK215, Fullmoon Biosystems, USA). The array contains 215 antibodies, each of which has 6 replicates, which has the unique capability of quantitative profiling of protein phosphorylation levels, by using paired unphospho- and phospho-antibodies for proteins. Protein microarray analysis was carried out using the protocol provided as described by Kang et al [42]. In brief, 40 μL of cell lysates in 70 μL of reaction mixture were labeled with 3 μL of biotin in 10 μg/μL *N,N*-dimethyformamide at room temperature for 2 h. After being treated with blocking solution for 45 min, the array was immersed in 6 mL of Coupling Mix for 2 h on a shaker at 35 rpm. The array was washed thoroughly and incubated with 30 mL of Cy3-conjugated streptavidin. Once rinsing with Milli-Q grade water, the array was dried and scanned with the GenePix 4000B (Axon Instruments, Foster city, USA). For each antibody, we computed the phosphorylation ratio.

### Quantitative real-time PCR

Total RNA was extracted from cell lines by TRIzol (MK3, Thermo Fisher Scientific, Massachusetts, USA) methods, and then was reverse transcribed as cDNA by using Reverse Transcription System (MK3, Thermo Fisher Scientific, Massachusetts, USA). The primers were designed using Primer 5.0. Quantitative real-time PCR (qRT-PCR) was performed using the SYBR Green qPCR (TOYOBO, Osaka Prefecture, Japan) for studying the quantitative expression of STC1, IκBα and AKT in accordance with the manufacturer's protocol. The relative expressions of mRNAs were normalized to those of internal reference β-actin, and were calculated by the 2^−ΔΔCt^ method. The following primers were used: STC1, forward 5′-GAAAGCTTATGCTCCAAAACTCAG-3′ and reverse 5′-TTCTCGAGTTATGCACTCTCATGG-3′; IκBα, forward 5′-GCGGATCCATGTACCAGGCGGC-3′ and reverse 5′-ACGCTCGAGACTCACCAGACGCT-3′; AKT, forward 5′-GAGGAGCGGGAAGAGTG-3′ and reverse 5′-GAGACAGGTGGAAGAAGAGC-3′; β-actin, forward 5′-AGGGGCCGGACTCGTCATACT-3′ and reverse 5′-GGCGGCACCACCATGTACCCT-3′.

### Western blotting analysis

Protein extracts from HeLa and CaSki cells were prepared using RIPA Lysis buffer (Auragene, Changsha, China) according to the manufacturer's protocol. The concentrations of extracted proteins were detected in accordance with the Bradford Protein Assay Reagent (Beyotime, Shanghai, China), and bovine serum albumin was utilized as a standard. Comparable amounts of protein samples were isolated using SDS-PAGE, and then proteins were transferred onto a PVDF membrane (Millipore, Bedford, USA). The PVDF was blocked with 5% non-fat milk in TBST buffer for 2 h at room temperature, and then was incubated over-night with the primary antibodiy for STC1 (1:1000, Abcam, Cambridge, UK), NF-κB phospho-P65 (Ser468, 1:500), IκBα (1:1000), IKKβ (1:1000), AKT (1:1000), PI3K(1:1000); phospho-IκBα (Ser32/36, 1:1000, Cell Signaling, Danvers, USA), NF-κB P65 (1:1000), PKC α/β II (1:1000), phospho-PKCα/β (Thr638, 1:1000), Bax (1:1000) and Bcl-2 (1:1000); NF-κB phospho-P65 (Ser536, 1:1000, Immunoway, Newark, USA), phospho-AKT (Thr308, 1:500), phospho-JNK1 (Thr183/Tyr185, 1:500); NFκB phospho-P65 (Thr254, 1:200, Santa Cruz, Texas, USA), NF-κB phospho-P65 (Ser311, 1:200), NF-κB (Thr435, 1:200), phospho-PI3K (1:200) and JNK1 (1:200); phospho-IKKβ (Tyr188, 1:1000, Yubo, Shanghai, China). Beta-actin antibody was acted as a control (1:1000, Auragene, Changsha, China), followed by incubation for 1 h with a suitable secondary antibody (Auragene, Changsha, China). Electrochemiluminescence was performed with a Gel Documentation and Analysis System (image Pro-plus 6.0 Media Cybernetics, Maryland, USA).

### Immunocytochemical staining for NFκB-phospho-P65

Cells were harvested with ice-cold PBS and fixed in ice-cold acetone for 20 min, and then the cells were quenched by 3% H_2_O_2_ in methanol for 15 min. After blocking in a serum-free protein block for 15 min, phospho-P65 antibody was added at a dilution of 1:50 and incubated with the samples for 1 h at 37°C, followed by detection with Dako Envision + HRP Labeled Polymer for 20 min, followed by incubation with chromogen DAB+ for 5 min.

### Statistical analysis

All statistical tests were conducted with SPSS 17.0 software (SPSS, Inc., Chicago, IL, USA). The variance between two groups and multiple groups was compared by t-test and ANOVA, respectively. The data were expressed as mean ± standard deviation (SD). A *p-*value < 0.05 was regarded as significant.

## SUPPLEMENTARY FIGURES AND TABLES


