# Supplementary material for: STC1 promotes cell apoptosis via NF-κB phospho-P65 Ser536 in cervical cancer cells
Source: Oncotarget. 2017 May 5;8(28):46249–61. doi: 10.18632/oncotarget.17641 (PMC5542264; doi:10.18632/oncotarget.17641)
Supplement: Supplementary file 1 [file oncotarget-08-46249-s001.pdf]

STC1 promotes cell apoptosis via NF-κB phospho-P65 Ser536 in Cervical cancer cells

SUPPLEMENTARY MATERIALS

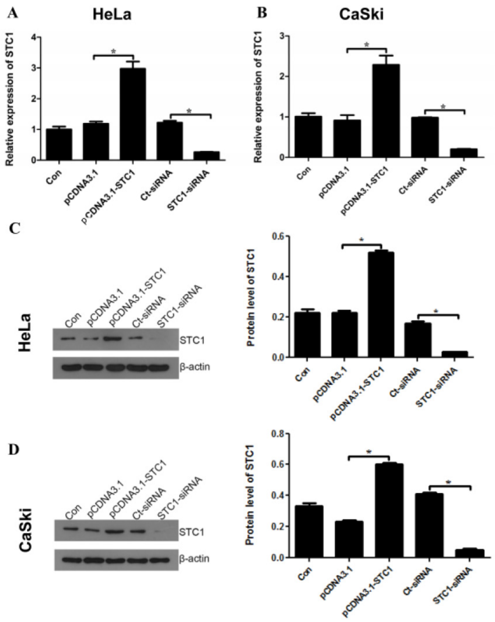

Supplementary Figure 1: The expression of STC1 in cervical cancer cells. mRNA expression of STC1 gene in STC1 overexpressed and inhibited cervical cancer HeLa **(A)** and CaSki **(B)** cells. Cell lysates were analyzed with Western blotting for STC1 in STC1 overexpressed and inhibited cervical cancer HeLa **(C)** and CaSki **(D)** cells. n=3, *p<0.05.

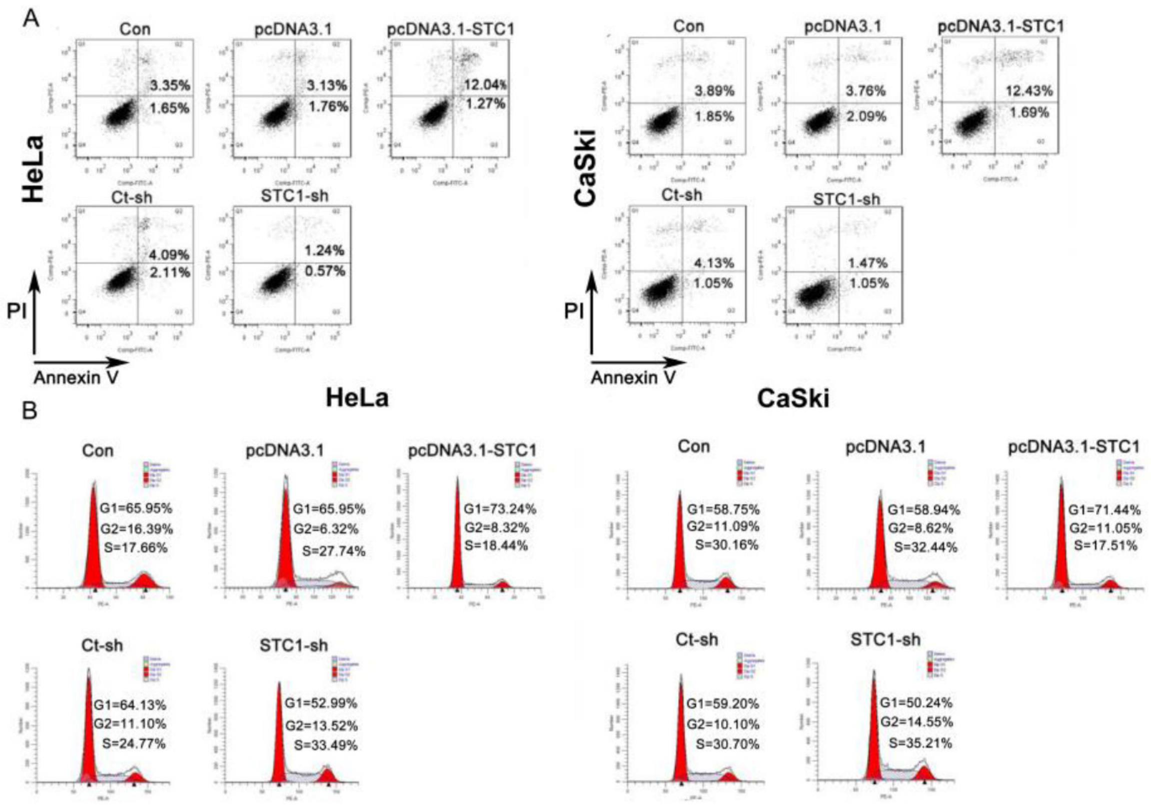

Supplementary Figure 2: Overexpression of STC1 promoted apoptosis and cell cycle in cervical cancer cells. (A) Annexin V-FITC /PI staining shows STC1 promoted cell apoptosis in cervical cancer HeLa and CaSki cells. **(B)** Flow cytometry analysis reveals STC1 arrested G1 phase of cell cycle in cervical cancer HeLa and CaSki cells. n=3, **p*<0.05.

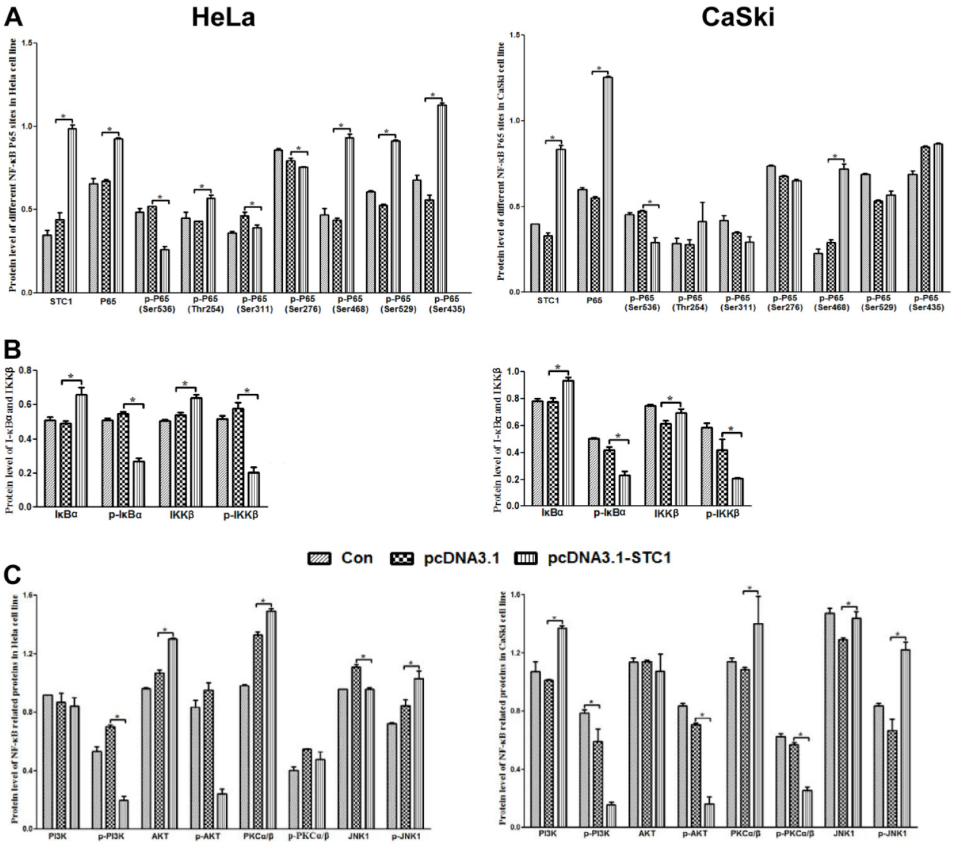

Supplementary Figure 3: The histogram of protein and phosphorylation levels of essential proteins in STC1 overexpressed cervical cancer cells. **(A)** The histogram of protein levels of NF-κB P65 and its exact phosphorylation site in STC1 overexpressed cervical cancer HeLa and CaSki cells (Western blotting). **(B)** The histogram of protein and phosphorylation levels of IκBα and IKKβ in STC1 overexpressed cervical cancer HeLa and CaSki cells (Western blotting). **(C)** The histogram of protein levels of PI3K, AKT, PKCα/β and JNK1 in STC1 overexpressed cervical cancer HeLa and CaSki cells (Western blotting). n=3, *p<0.05.

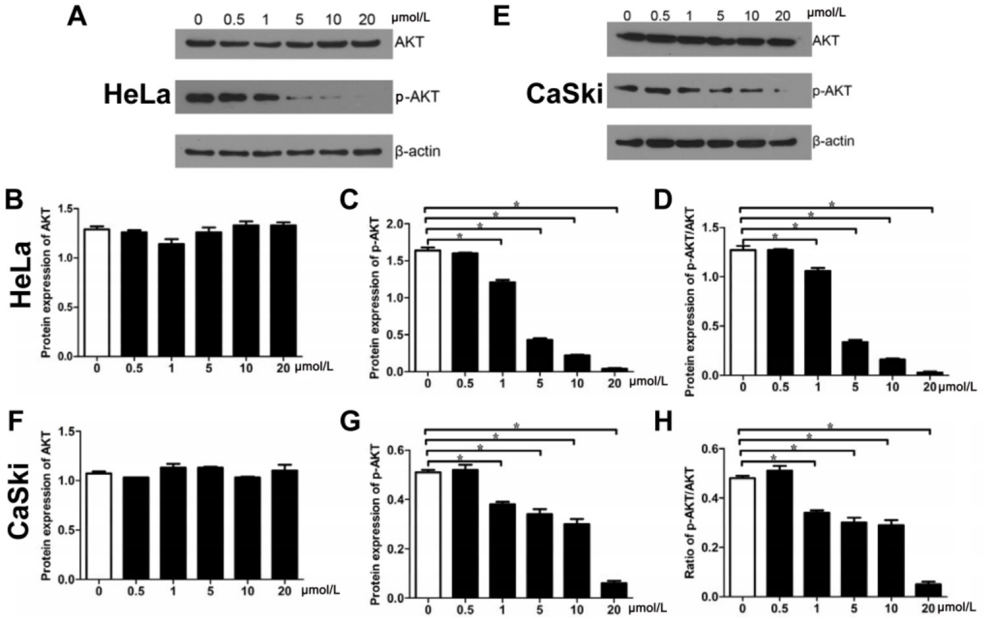

Supplementary Figure 4: The protein and phosphorylation levels of AKT in cervical cancer cells treated with LY294002. Western blotting reveals similar level of AKT expression and different level of phospho-AKT in cervical cancer HeLa **(A-D)** and CaSki cells **(E-H)** treated with PI3K inhibitor LY294002 (0, 0.5, 1, 5, 10, 20 μmol/L). n=3, **p*<0.05.

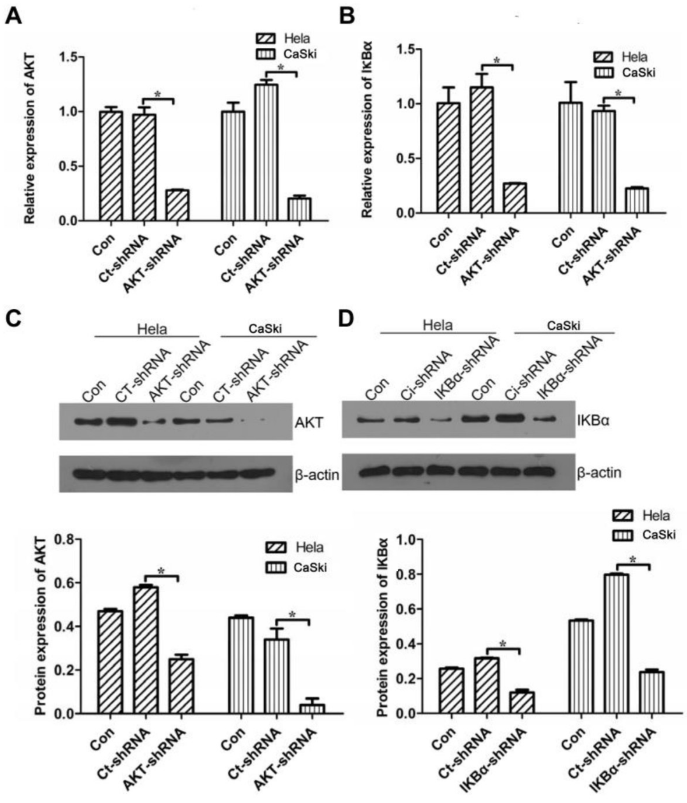

Supplementary Figure 5: The expression of AKT and IκBα in cervical cancer cells. Quantitative Real-time PCR validation of AKT **(A)** gene and IκBα **(B)** gene in AKT-inhibited cervical cancer HeLa and CaSki cells. **(C)** Western blotting shows the protein of AKT in AKT-inhibited cervical cancer cells. **(D)** Western blotting reveals the protein of IκBα in IκBα-inhibited cervical cancer cells. n=3, *p<0.05.

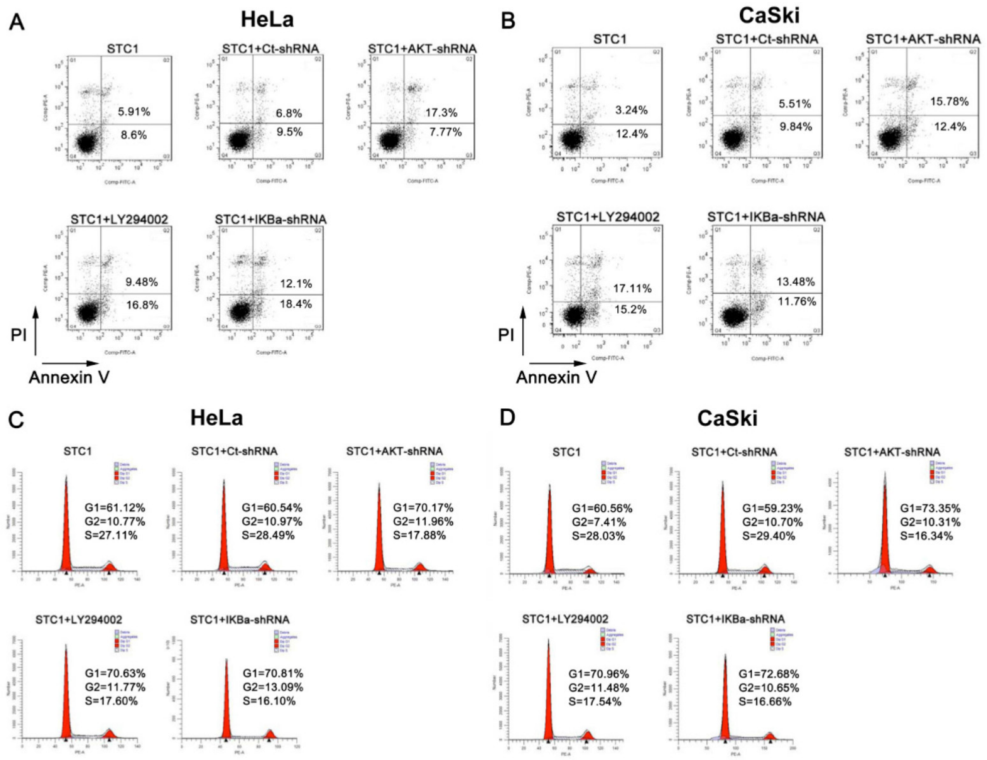

Supplementary Figure 6: AKT, PI3K and IκBα inhibited apoptosis and promoted G1/S transition in STC1 overexpressed cervical cancer cells. **(A)** Annexin V-FITC/PI staining shows the inhibition of AKT (AKT-shRNA), PI3K (PI3K inhibitor LY294002) and IκBα (IκBα-shRNA) promoted the cell apoptosis of cervical cancer HeLa **(A)** and CaSki **(B)** cells. n=3, **p*<0.05. **(B)** Annexin V-FITC /PI staining reveals the AKT-shRNA, PI3K inhibitor LY294002 and IκBα-shRNA suppress the S phase of cervical cancer HeLa **(A)** and CaSki **(B)** cells. n=3, **p*<0.05.

Supplementary Table 1: Correlation between STC1 expression and clinicopathlogical features in patients with cervical cancer

	STC1 low expression	STC1 high expression	*p* value
Age			*p*=0.237
<45	16	19	
>45	20	15	
Stage			*p*=0.034
I-II	19	26	
III	17	8	

Supplementary Table 2: Fold change of phospho-protein levels in STC1-overexpressed cells were chosen from phospho-antibody microarray

Name	CaSKi NC-phos/unphos	CaSKi STC1-phos/unphos	CasKi-STC1/CasKi-NC	Swiss prot
AKT (Phospho-Thr308)	1.65	1.37	0.83	P31749
CK2-b (Phospho-Ser209)	1.57	1.34	0.86	P67870
COT (Phospho-Thr290)	0.98	0.90	0.91	P41279
IκB-alpha (Phospho-Ser32/36)	2.34	1.21	0.52	P25963
IKK beta (Phospho-Tyr188)	1.10	0.87	0.80	O14920
JNK1/2/3 (Phospho-Thr183/Tyr185)	1.17	0.96	0.82	P45983/P45984/P53779
NFκB-P65 (Phospho-Ser276)	0.78	0.96	1.23	Q04206
NFκB-P65 (Phospho-Ser311)	5.83	8.41	1.44	Q04206
NFκB-P65 (Phospho-Ser468)	1.33	1.78	1.34	Q04206
NFκB-P65 (Phospho-Ser529)	1.46	2.12	1.45	Q04206
NFκB-P65 (Phospho-Ser536)	2.43	2.17	0.89	Q04206
NFκB-P65 (Phospho-Thr254)	0.76	0.66	0.87	Q04206
NFκB-P65 (Phospho-Thr435)	2.00	2.01	1.00	Q04206
PI3-kinase p85-subunit alpha/gamma (Phospho-Tyr467/Tyr199)	0.91	1.02	1.13	P27986/Q92569
PKC alpha/beta II (Phospho-Thr638)	2.37	1.41	0.59	P17252
PKR (Phospho-Thr446)	2.40	2.39	0.99	P19525
PKR (Phospho-Thr451)	0.92	1.17	1.28	P19525
TAK1 (Phospho-Thr184)	0.52	0.64	1.24	O43318

Alteration of phosphorylation sites in NF-κB signaling. Analysis of the Phospho-protein profiling in CaSki/STC1 cells and CaSki/NC cells via phospho-protein antibody array designed for NF-κB signaling pathway. The apoptosis-related proteins included AKT, IκBα, IKK, JNK, P65, PI3K, CK2, COT, PKR and TAK1.

Supplementary Table 3: Forward and reverse primer of candidate shRNA targets sequence

Primer	Sequences
STC1	F: 5'-GATCC TTAGTCCAGGAAGCAATAGTA TTCAAGAGA TACTATTGCTTCCTGGACTAA TTTTTTA-3'
	R: 5'-GATCC AATCAGGTCCTTCGTTATCAT TTCAAGAGA ATGATAACGAAGGACCTGATT TTTTTTA-3'
IκBα	F: 5'-GATCC GGAGTGTTAAGCGTTCAGTGA TTCAAGAGA TCACTGAACGCTTAACACTCC TTTTTTA-3'
	R: 3'-G CCTCACAATTCGCAAGTCACT AAGTTCTCT AGTGACTTGCGAATTGTGAGG AAAAAATTCGA-5
AKT	F: 5'-GATCC GGACTACCTGCACTCGGAGAA TTCAAGAGA TTCTCCGAGTGCAGGTAGTCC TTTTTTA-3'
	R: 3'-G CCTGATGGACGTGAGCCTCTT AAGTTCTCT AAGAGGCTCACGTCCATCAGG AAAAAATTCGA-5

Construction of STC1-shRNA vector, AKT-shRNA vector and IκBα-shRNA vectors. The forward and reverse primer sequences of candidate targets are shown as Supplementary Table 3. One negative control shRNA is named Ct-sh.
